# A case study of adapting a health insurance decision intervention from trial into routine cancer care

**DOI:** 10.1186/s13104-022-06189-8

**Published:** 2022-09-10

**Authors:** Miles E. Charles, Lindsay M. Kuroki, Ana A. Baumann, Rachel G. Tabak, Aimee James, Krista Cooksey, Mary C. Politi

**Affiliations:** 1 Division of Public Health Sciences, Department of Surgery, Washington University School of Medicine, 660 S. Euclid Ave, Campus, Box 8100, St. Louis, MO 63110 USA; 2 Division of Gynecologic Oncology, Department of Obstetrics and Gynecology, Washington University School of Medicine, St. Louis, USA; 3 Brown School, Washington University, St. Louis, USA; 4 Prevention Research Center, Washington University, St. Louis, USA

**Keywords:** Implementation, Adaptation, Decision science, Health insurance, Health policy

## Abstract

**Objective:**

This study adapted *Improving Cancer Patients’ Insurance Choices* (*I Can PIC),* an intervention to help cancer patients navigate health insurance decisions and care costs. The original intervention improved knowledge and confidence making insurance decisions*,* however, users felt limited by choices provided in insurance markets. Using decision trees and frameworks to guide adaptations, we modified *I Can PIC* to focus on *using* rather than *choosing* health insurance. The COVID-19 pandemic introduced unforeseen obstacles, prompting changes to study protocols. As a result, we allowed users outside of the study to use *I Can PIC* (> 1050 guest users) to optimize public benefit. This paper describes the steps took to conduct the study, evaluating both the effectiveness of *I Can PIC* and the implementation process to improve its impact.

**Results:**

Although *I Can PIC* users had higher knowledge and health insurance literacy compared to the control group, results were not statistically significant. This outcome may be associated with systems-level challenges as well as the number and demographic characteristics of participants. The publicly available tool can be a resource for those navigating insurance and care costs, and researchers can use this flexible approach to intervention delivery and testing as future health emergencies arise.

**Supplementary Information:**

The online version contains supplementary material available at 10.1186/s13104-022-06189-8.

## Introduction

JL is a 66-year-old patient with progressive, recurrent ovarian cancer whose clinician recommended that she start on a targeted, oral cancer therapy based on genomic testing of her cancer. A month after receiving this recommendation, JL received a “summary of benefits” from her insurance company reflecting she owed a $3000 USD co-pay for a 30-day supply of this targeted therapy (the goal was to continue this therapy until her disease no longer responded to it, or she had intolerable side effects; her clinicians estimated this might take 1–2 years). As a full-time employed nurse, JL had health insurance. However, she did not qualify for the industry-sponsored financial assistance drug program because her annual income was slightly ($3500) over the allowed threshold. She would have to spend down 3% of her income on prescriptions that year in order to receive 100% coverage for the medication. Furthermore, because she had both government-sponsored and private insurance, her government-sponsored insurance made her ineligible for a “co-pay card” sponsored by the pharmaceutical company. JL was extremely distressed about this financial strain and considered whether and how she could take this therapy recommended by her doctor.

JL, like many under-insured patients, was inadvertently overlooked by her oncology team to be at risk for what scholars refer to as “financial toxicity,” or the material and psychosocial hardship from high costs of care. Yet, as many as 64% of patients report financial hardship following a cancer diagnosis [1], and many face barriers, like those described above, that prohibit affordable access to needed cancer therapies [2, 3]. We use this case study to describe the critical steps we took to adapt and implement a health insurance decision intervention for cancer patients and survivors like JL, while balancing intervention testing and adaptation with real-world needs during a global pandemic.

## Main text

### Evidence supporting the intervention and the need for adaptation

*Improving Cancer Patients’ Insurance Choices* (*I Can PIC)* is an interactive online decision tool originally designed to help cancer patients, like JL, think through their health insurance choices and identify ways to offset high costs of cancer and survivorship [4]. It provides tailored cost estimates across insurance plan types based on demographic and health characteristics and provides financial support resources.

In a randomized controlled trial of *I Can PIC* compared to an attention control group where participants were given an alternative intervention: a handout that lists financial resources along with brief definitions of health insurance terms, *I Can PIC* users knew more about health insurance and were more confident understanding insurance terms [4]. However, many *I Can PIC* users reported that their employer-based and marketplace insurance gave them limited choices [4]. This implied the potential to better align the tool within the current insurance landscape, even if it required adaptation before meeting all of its goals [5]. Therefore, the team elicited feedback from clinicians, patients, and policy experts on ways to emphasize *using* health insurance rather than focusing mostly on *choosing* health insurance (Additional file 2: Table S1). This paper describes the adaptation process of *I Can PIC* to achieve these goals.

### Methods

#### Intervention adaptation process

**Fig. 1:**
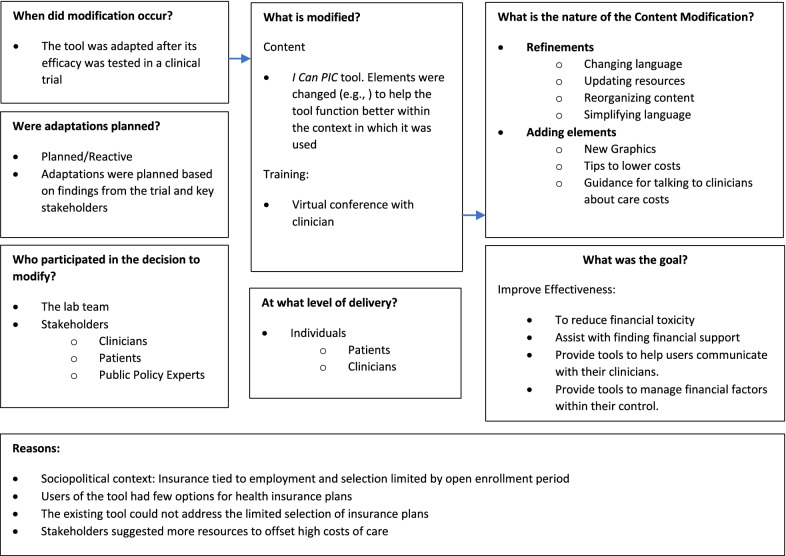
*I Can PIC* as Tracked and Adapted Using the FRAME Approach

We used two guides to structure the adaptation process. The Iterative Decision-making for Evaluation of Adaptations (IDEA) decision tree informed the process of adaptation [6], and the Framework for Reporting Adaptation and Modifications-Expanded (FRAME) guided the tracking of adaptations (Fig. 1) [7]. To start the adaptation process, we first identified the core elements of the intervention that improved outcomes: health insurance educational resources, cost-of-care conversation guidance, and resources to offset costs which are critical to patients like JL (Additional file 1: Fig S1). During this iterative process, we then added new elements to *I Can PIC* and made content, format, and functional improvements based on stakeholder feedback and the original trial results (Additional file 2: Table S1).

#### Assessment of the adapted I Can PIC tool

After we adapted *I Can PIC*, we assessed its effectiveness among newly-diagnosed patients with gynecological, colorectal, or lung cancer, examining their health insurance knowledge, financial toxicity, health insurance literacy, and delayed or forgone care due to cost. We conducted a historic control survey assessing these constructs as well as whether and how treatment costs were discussed with patients at their most recent visit with their physicians. Next, we conducted a brief virtual/video conference training with fifteen medical or surgical oncologists to talk about screening for financial distress and discussing costs with patients. After the training, we conducted a pilot intervention study where patients were sent *I Can PIC*, completed a survey after their upcoming oncology appointment, and a follow-up survey 3–6 months after recruitment. Once during the study, we gave the oncologists feedback and reminded them to screen for financial distress and refer patients to *I Can PIC.* This study was approved by the Human Research Protection Office at Washington University in St. Louis (protocol number 202003033).

The COVID-19 pandemic introduced unforeseen obstacles to patient enrollment that shifted in-person recruitment to virtual methods (e.g., phone calls and emails) from secured, Health Insurance Portability and Accountability Act (HIPAA) compliant university-affiliated phone numbers and emails. We also partnered with the institutional review board and streamlined the consent script to be more succinct and engaging [8]. Prior to these changes and even after, many newly diagnosed cancer patients did not want to add a research commitment to their already busy or overextended lives. With recognition of these challenges and others, the revised version of *I Can PIC* was made available to the public while undergoing testing so that patients outside of eligibility criteria, like JL with recurrent cancer, or patients not interested in research, could still benefit from its health insurance and care costs information and support.

#### Procedure

Eligible patients for both the historic control and intervention groups were English speaking, at least 18 years old, eligible for insurance through their employer or the federal marketplace, and diagnosed with a new lung, gynecological, or colorectal cancer within five months. Participants were recruited from a single site, NCI-designated cancer center where fifteen oncologists (five gynecologic oncologists, three colorectal surgeons, two medical oncologists treating colorectal cancer patients, and five thoracic surgeons treating lung cancer patients) gave the study team permission to review medical records and approach eligible patients for study participation. Recruitment into the historic control group began in May 2020. Starting in October 2020, we conducted our first provider training, since health insurance open enrollment was beginning, and we wanted at least some patients to use *I Can PIC* while they had options of changing insurance. Between October 2020 and February 2021, we trained the fifteen oncologists to screen for financial toxicity and discuss care costs with patients.

After clinicians were trained, we recruited patients into the intervention arm. Patients were asked to review *I Can PIC* before their upcoming appointment. After they met with their oncologist, the research team sent them a survey that could either be completed online or by phone. A three-month follow-up survey was also sent to patients in the intervention arm.

#### Measures

Patient socio-demographics were self-reported. As in the original trial, participant numeracy and health literacy were assessed using validated scales [9–12]. Primary outcomes included health insurance knowledge, health insurance literacy, frequency, and type of care cost conversations (including topics and strategies discussed), financial toxicity, and patient referrals to resources to further discuss costs [13].

#### Statistical analysis

Descriptive statistics were calculated for all sociodemographic variables and compared between groups using chi‐square analyses or Fisher's exact test for categorical variables, as appropriate, and the Kruskal–Wallis test for continuous variables. Baseline surveys for both the control and intervention groups were compared for one-way analysis of variance, Fisher's exact test to determine if there were nonrandom associations between two categorial variables, and Chi-Square tests to determine if two categorical variables were independent. To compare the intervention at the baseline survey and 3–6 month follow-up survey, paired t-tests for continuous variables and kappa statistics for categorical variables (discussed costs or not, discussed cost strategies or not, referral made or not, etc.)

### Results

During our study period, there were 1512 total logins on the *I Can PIC* website, of which 1058 (70%) were guest users. Guest users were treated in other facilities, ineligible due to cancer type, or not interested in participating in the research study, but wanted to access the information. Among the 136 consented and surveyed participants (68 historic controls; 68 intervention group), socio-demographics were similar except that the intervention group was slightly higher educated (Table 1). The intervention group had slightly higher health insurance knowledge (mean score 77.02 vs 72.45) and slightly higher health insurance literacy (mean score 34.71 vs 33.03) compared to controls; these differences were not statistically significant. Knowledge and health insurance literacy was sustained at the 3–6 month follow-up.

**Table 1:** Participant demographics and cost conversations

	Historical Control (n = 68)	Intervention (n = 68)
Mean Age	60.49	58.57
Race		
Black/African American	4 (5.88%)	3 (4.41%)
Caucasian/White	58 (82.29%)	65 (95.59%)
Other	6 (8.82%)	0 (0.00%)
Mean Household income	$70,000	$67,500
Education		
HS diploma or less	22 (32.35%)	15 (22.06%)
Some College	15 (22.06%)	14 (20.59%)
College degree or more	28 (41.18%)	39 (57.35%)
Missing	3 (4.41%)	
Health Literacy (SILS)		
Adequate	52 (76.47%)	56 (82.35%)
Limited	13 (19.12%)	12 (17.65%)
Missing	3 (4.41%)	0 (0.00%)
Cost Strategies		
1. Changing logistics of care	21 (30.88%)	18 (26.47%)
2. Setting up a payment plan	2 (2.94%)	12 (17.65%)
3. Changing the dose of your treatments	7 (10.29%)	8 (11.76%)
4. Choosing a generic drug	6 (8.82%)	8 (11.76%)
5. Referring to a hospital billing office	8 (11.76%)	5 (7.35%)
6. Looking for copay assistance, coupons, rebates	2 (2.94%)	6 (8.82%)
7. Stopping or pausing some treatments	4 (5.88%)	3 (4.41%)
8. Other	4 (5.88%)	1 (1.47%)
9. Suggesting a community agency or charity	2 (2.94%)	2 (2.94%)
10. Suggesting a free drug program	1 (1.47%)	–
11. Suggesting you talk to human resources	1 (1.47%)	–
12. Choosing a lower cost procedure	0 (0.00%)	0 (0.00%)
13. Suggesting government assistance	0 (0.00%)	0 (0.005)

The frequency of cost discussions related to cancer care was similar between the intervention and control groups (57.4 vs 67.7%, p = 0.22), with the most common topics involving insurance, time off work, and costs of medications. Specific cost strategies that were discussed are detailed in Table 1. Overall, a small proportion of patients received referrals (eg., *I Can PIC* website or any outside agency/office such as government assistance, community agency or charity, or hospital billing) from their oncologist to learn more about cancer costs and did not vary by group (controls, 16.2% vs intervention group, 20.6%).

Financial toxicity was reportedly low in both groups (17.7% in the control vs 16.1% in the intervention group), though decreased slightly within the intervention group during the study period (first survey average score was 16.06 vs. 14.17 at the 3–6 month follow-up). Unfortunately, 18% of individuals in the control group and 13% in the intervention group reported delaying care due to cost (p = 0.41).

## Outlook

### Discussion

Throughout the adaptation process, it is important to ensure that end users like JL can benefit from effective interventions, even if interventions require refinement and continued testing. Using systematic decision trees and guides such as IDEA and FRAME, we described one way to systematically track intervention adaptations while ensuring real-world access throughout the process to benefit patients. Strengths of our study include our diverse stakeholders which included patients, clinicians and policy experts who provided advice on *I Can PIC*, including patients across several cancer types, and modification of consent processes and tool access to optimize patient engagement and minimize burden.

This case example provides a guide for deploying low-risk interventions in routine care while continuing to generate evidence and improve on their public health impact. Of 1512 total logins on the *I Can PIC* website, 70% were guest users outside of the research study, and we hope many of them, like JL, benefited from *I Can PIC* access even if they were reluctant to join a study. JL ultimately made an informed decision with her oncology team and the support of her family to only work part-time to optimize her benefits in order to receive the targeted, oral cancer therapy through the pharmaceutical company’s financial assistance program.

Despite growing awareness of financial toxicity on underinsured patients, more interventions are needed to better integrate cost conversations into routine cancer care. Systems-level changes are needed to address this burden of care on patients. Future work will continue to build on the frameworks discussed to adapt content and delivery of *I Can PIC* so that patient-centered outcomes, such as financial toxicity, distress, quality-of-life, and adherence to treatment, are improved. This case study can provide guidance for other implementation studies, including those that might be conducted during future health emergencies.

### Limitations

Due to rapid changes as a result of the COVID-19 pandemic, it is important to note limitations of our study design and execution of our protocol. Given this unprecedented time when unmet social and health needs were and still remain under constant threat and turmoil, we acknowledge our non-randomized study design and recruitment of historic controls are critical limitations to interpretation and generalizability of results. The timing of their recruitment could have exacerbated health or financial strain, although the pandemic was still ongoing even at the end of the study with new waves of health risks emerging. Future health emergencies could introduce similar issues without addressing the larger social and societal needs. Furthermore, COVID-19 and rapid transitions to virtual recruitment presented other challenges to this project, which was initially planned to be in person. Despite modifications to the protocol, consent documents, and workflow to reduce burden on participants, systemic issues remained that reduced the diversity of our sample in the research component of intervention implementation. These challenges are likely to remain without addressing systemic barriers to research and care more broadly. Consequently, these results may not be representative of the experiences of lower income and/or racially diverse patients experiencing financial toxicity due to their cancer diagnosis. Ongoing feedback from stakeholders will continue to ensure that the needs of various populations, including oncology providers are considered.

## Supplementary Information

**Additional file 1: Figure S1.** *I Can PIC *as Adapted and Tracked Using the Iterative Decision-Making for Evaluation of Adaptations (IDEA) Framework Steps.

**Additional file 2: Table S1.** Stakeholder- and Participant-Suggested Adaptations to *I Can PIC.*

## Data Availability

The datasets used during the current study are available from the corresponding author on reasonable request.

## Abbreviations

I Can PIC: Improving Cancer Patients’ Insurance Choices
IDEA: Iterative Decision-making for Evaluation of Adaptations
FRAME: Framework for Reporting Adaptation and Modifications-Expanded
HIPAA: Health Insurance Portability and Accountability Act
