# Supplementary material for: A case study of adapting a health insurance decision intervention from trial into routine cancer care
Source: BMC Res Notes. 2022 Sep 10;15:298. doi: 10.1186/s13104-022-06189-8 (PMC9463661; doi:10.1186/s13104-022-06189-8)
Supplement: Supplementary file 1 — Additional file 1: Figure S1. I Can PIC as Adapted and Tracked Using the Iterative Decision-Making for Evaluation of Adaptations (IDEA) Framework Steps. [file 13104_2022_6189_MOESM1_ESM.docx]

Additional file Figure S1 : *I Can PIC* as Adapted and Tracked Using the Iterative Decision-Making for Evaluation of Adaptations (IDEA) Framework Steps

1. Is Adaptation Needed?
   - The Clinical Trial of *I Can PIC* indicated that users had limited options for health insurance plans. As a result of this finding, the research team and stakeholders decided to adapt the tool so that it could address financial concerns within the limitations of insurance choice.
2. Are core elements or core functions of the intervention known?
   - The function of the original elements of *I Can PIC* were known, however, they were not as effective in a practical context, as users had limited health insurance options.
3. Can concerns be addressed while preserving core intervention element?
   - To address the deficits in outcomes, the research team emphasized elements that allowed users to reduce costs within their limited choices, and then moved the health insurance plan choice section to the end for those users who had choices during open enrollment.
4. Does timeframe allow pilot?
   - The adapted tool can be tested in an ongoing implementation study at a major cancer treatment center.

E/F. Are desired outcomes improved? Is voltage drop acceptable?

- Desired outcomes will be assessed in the ongoing study.
