# Supplementary material for: A case study of adapting a health insurance decision intervention from trial into routine cancer care
Source: BMC Res Notes. 2022 Sep 10;15:298. doi: 10.1186/s13104-022-06189-8 (PMC9463661; doi:10.1186/s13104-022-06189-8)
Supplement: Supplementary file 2 — Additional file 2: Table S1. Stakeholder- and Participant-Suggested Adaptations to I Can PIC. [file 13104_2022_6189_MOESM2_ESM.docx]

| Additional file Table S1 : Stakeholder- and Participant-Suggested Adaptations to *I Can PIC* | | |
| --- | --- | --- |
| Type of Adaptation | Examples | Stakeholders involved |
| Organizational Edits | Numerous organizational edits where made to focus on health insurance education, cost conversations and financial resources before plan selection. Examples include:   - Changing the order of the tool sections - Subdividing the “Let’s Learn” section of the tool to have separate focused conversations about health insurance education and healthcare costs - Streamlining the new sections of the “Let’s Learn page” | Research team  Patient and community stakeholders  Trial participants |
| Additions to the tool | Many new elements were added to the tool based on stakeholder feedback to better address participant needs. These include:   - Additional health insurance knowledge items, such as an explanation of Health Savings Accounts - Guidance on how to read and interpret a hospital bill with sample bills - Guidance on how to read an explanation of benefits (EOB) - Tips for keeping track of bills - Tips for identifying and rectifying billing errors or surprise bills - Additional local, regional, and national resources to help offset care costs | Patient and community stakeholder and trial participants, external scientific stakeholders. Feedback was collected through surveys and phone interviews |
| Removal of confusing information | - Updated terminology and clarified language about difficult words - Revisions to title, subheadings and texts of the pages of the tool | Research team, external scientific stakeholders, community organizations |
